# Aqueous Dispersion of Unmodified Fullerene C60: Stimulation of Hair Growth and Study of a New Molecular Target for Interaction

**DOI:** 10.3390/ijms26178517

**Published:** 2025-09-02

**Authors:** Nadezda Shershakova, Elena Baraboshkina, Dmitry Khochenkov, Evgeny Turetskiy, Alexandra Nikonova, Oleg Kamyshnikov, Daria Bolyakina, Veronika Parshina, Daria Shabanova, Evelina Makarova, Sergey Andreev, Dmitry Kudlay, Musa Khaitov

**Affiliations:** 1 NRC Institute of Immunology FMBA of Russia, 115522 Moscow, Russia; 2 FSBI “N.N. Blokhin National Medical Research Center for Oncology”, Ministry of Health of the Russian Federation, 115522 Moscow, Russia; 3 Department of Pharmaceutical and Toxicological Chemistry Named After A.P. Arzamastsev, FSAEI HE I.M. Sechenov First MSMU of MOH of Russia (Sechenovski University), 119435 Moscow, Russia; 4 Laboratory of Molecular Biotechnology, Mechnikov Research Institute for Vaccines and Sera, 105064 Moscow, Russia; 5 Department of Immunology, FSAEI HE N.I. Pirogov RNRMU MOH Russia, 117997 Moscow, Russia

**Keywords:** fullerene C60, hair growth, Wnt10b, adenosine A_2A_ receptor, M2 macrophages

## Abstract

Hair loss (alopecia) is a common disorder caused by an interruption in the body’s cycle of hair production. This pathology negatively affects the psychoemotional state of patients and significantly reduces their quality of life. The currently available medical treatments (including minoxidil therapy) are effective in arresting the progression of the disease; however, they allow only partial regrowth of hair at best. A significant clinical result occurs only with regular drug use. There is still great interest in finding new drugs for the treatment of alopecia. In this study, we aimed to examine the effect of an aqueous dispersion of unmodified fullerene C60 (ADF) on hair growth. ADF, produced by a unique technology, is biocompatible and non-toxic. Nu/nu mice were subcutaneously injected (2 μg/animal) every two days for a period of 11 days with ADF and, for control purposes, with phosphate-buffered saline (PBS). It was shown that ADF stimulated hair growth. Histological analysis of the nu/nu mice skin areas showed that animals treated with ADF had significantly more (about twice as many) hair follicles in the anagen phase compared to mice treated with PBS. The effect on hair growth persisted even after discontinuation of ADF administration. Analysis of gene expression demonstrated that ADF affected the Wnt-signaling pathway, increased the expression of the *Wnt10b* (wingless-type Mouse Mammary Tumor Virus integration site family, member 10B) factor, angiogenetic factors, and downregulated tumor necrosis factor-alpha levels. We propose that the mechanism of ADF action is likely related to its ability to attract macrophages to the hair follicle microenvironment and promote their polarization to the M2 phenotype. In addition, using molecular modeling, we tried to substantiate our hypothesis about the interaction of ADF with the adenosine A2A receptor, which may cause a decrease in tumor necrosis factor-alpha production. Thus, ADF may become a promising drug for the development of new approaches to the treatment of alopecia associated with immune disorders.

## 1. Introduction

Hair loss is a common disorder caused by an interruption in the body’s cycle of hair production. It can occur at any age; about 60% of patients with alopecia are under thirty years of age, and it affects both men and women [1,2,3]. Hair loss can be caused by a variety of factors, including genetics, hormones, medical conditions, and lifestyle choices. Additional causes of alopecia are multifactorial and include a variety of genetic and environmental factors, infections, surgery, hormonal disorders, drug application, and scalp trauma [4,5,6]. The most common types of alopecia include androgenetic alopecia, alopecia areata, telogen effluvium, and scarring alopecia. Alopecia areata is an autoimmune inflammatory disease, whereas androgenetic alopecia is androgen-dependent [7]. The cause of telogen effluvium is typically a physically or psychologically stressful event [8]. Hair loss can be caused by chemotherapy, radiation sickness, taking excessive amounts of medications, as well as skin diseases, cancer, hypothyroidism, viral, bacterial and fungal infections. Chemotherapy-induced alopecia (CIA) is probably one of the most shocking aspects for oncological patients and underestimated by physicians [9].

There are three periodic stages of hair growth—anagen (the growth phase), catagen (the transitional phase), and telogen (the resting phase)—that are controlled by the local signaling milieu and mediated by changes in several genes’ expression. The most important factor regulating growth and development of the hair follicles (HF) is WNT10B [10,11]. WNT10B overexpression induces the HF telogen phase to proceed into anagen earlier [12].

Among the many growth factors expressed in association with the development of hair follicles, epidermal growth factor (EGF) has been implicated in the control of epidermal and mesenchymal cell function, and it is likely that they also stimulate proliferation and differentiation of the cells of the cutaneous appendages during development [13]. Vascular endothelial growth factor A (VEGF-A) promotes the growth and migration of endothelial cells, inhibiting apoptosis and inducing angiogenesis and vasculogenesis [14]. It is known that tumor necrosis factor-alpha (TNFα) plays a major role in the pathogenesis of most types of alopecia. TNFα is synthesized in epidermal keratinocytes [15], and it is a potent proliferation inhibitor. It is known that the regulation of TNFα expression occurs due to the activation of the adenosine receptor A2A (ADORA2A), which in turn plays an important role in suppressing inflammation and protecting tissue from damage [16,17].

An important aspect in studying the activation mechanism of hair growth is the role of M2 macrophages. M2 macrophages reduce proinflammatory cytokine release and promote angiogenesis [18]. Macrophages are involved in the regulation of the hair growth cycle and also in tissue repair and regeneration through M1/M2 phenotype polarization [19]. The number of M1 around HF increases during catagen, while more M2 are found during anagen and telogen [20]. M2 macrophages reduce proinflammatory cytokine production and promote the activation of angiogenesis [18]. In addition, M2 can secrete various growth factors such as VEGF, EGF, and fibroblast growth factors (bFGFs)—which regulate various cellular processes, including proliferation, migration, and differentiation— and activates the Wnt/β-catenin signaling pathway, participating in HF regeneration and promoting neogenesis [21,22]. It should be noted that FGFs are a family of cell signaling proteins that are directly involved in hair follicle morphogenesis by regulating various cellular processes, including proliferation and migration [13]. bFGF induces angiogenesis and can promote the differentiation of hair follicle stem cells into endothelial cells [23,24].

The current therapies for alopecia include the use finasteride, minoxidil, topical prostaglandins, natural supplements, micro-needling, low-level laser light, platelet-rich plasma, fractional lasers, cellular therapy, and Wnt activators. Topical finasteride and minoxidil are the most widely used pharmacologic agents for alopecia therapy [25]. Finasteride is a potential teratogen and is generally contraindicated in women with childbearing potential while the efficacy of topical therapy is limited. Minoxidil is effective in alopecia by prolonging anagen, shortening telogen, and enlarging miniaturized follicles [26]. A significant clinical result occurs after 3–4 months and, in cases of minoxidil monotherapy, is maintained only with regular drug use.

Thus, given the growing relevance of the problem of hair loss, there is a need for new, effective, and safe drugs for the treatment of alopecia. In our opinion, the study of nanomaterials is a promising direction of research in this area. Nanomedicine is an actively developing branch of nanotechnology associated with the use of nanoparticles ranging in size from 1 to 100 nm for the treatment of various diseases. Nanostructures can be an alternative for alopecia treatment due to their long-lasting effects [27] and more favorable pharmacokinetic profile [28]. In our work, we attempted to evaluate the effectiveness of using fullerene C60 nanoparticles. Fullerenes have strong antioxidant activity and may have therapeutic potential for a wide range of diseases associated with oxidative stress, including alopecia [29,30,31,32,33,34,35,36]. Currently, hundreds of fullerene C60 derivatives have been synthesized that have a number of biological activities: antitumor [37], antiviral [38,39], antimicrobial [40], antioxidant [41,42], neuroprotective [43], photodynamic [44] effects, membranotropic [45], enzyme inhibitors, apoptosis blockers, and they may act as radioprotectors [46]. However, little is known about the molecular mechanisms of action of new nanostructures, in particular, fullerenes, on human cells. It is known that the fullerenes could affect the underlying mechanisms that initiate HF activation and hair growth [30], but the molecular mechanisms of this are unclear.

An important aspect in studying the biological activity of nanoparticles is the assessment of their toxicity. It should be noted that the data on the systemic toxicity of aqueous solutions of fullerene C60 are limited, however, no adverse effects have been reported with systemic or local administration [32,47,48]. It was shown that, in the organism, fullerene can be oxidized into fullerenol [49] and excreted through the bile ducts and kidneys. There are established pathways for fullerene C60 degradation in the environment. It has been observed in laboratory settings that, under UV irradiation, fullerenes readily oxidize into fullerenols [50], which can then be completely decomposed by basidiomycetes [51].

Thus, the purpose of the current study was to determine the ability of the ADF form to influence the intensity of hair growth, activate the Wnt-signaling pathway, and investigate the expression of genes associated with HF development. An important research task was to demonstrate the duration of the hair growth effect, because this question is relevant for alopecia treatment. In addition, in the context of studying the mechanism of action of ADF and searching for molecular targets, a hypothesis was put forward about the ability of ADF to attract and activate macrophages, as well as a hypothesis about the interaction of fullerene C60 with ADORA2A.

## 2. Results and Discussion

### 2.1. ADF Stimulates Hair Growth

The aqueous dispersion of unmodified fullerene C60 at high concentration was produced by a unique scalable technology using «green technology» [32]. An important property of the obtained ADF is its low toxicity, biocompatibility, and safety via different administration routes [32,52].

In the current study, we evaluated the effect of topical application and subcutaneous injections of unmodified fullerene C60 aqueous dispersion in shaved BALB/c mice on hair growth. After the last s.c. injections and e.c. applications of ADF, pictures of the BALB/c mice back areas were taken (Figure S1). Visual assessment of the mice’s skin after topical treatment with ADF or PBS showed that, in the group treated with ADF, the intensity of hair growth was noticeably higher compared to the control groups treated with PBS (Figure S1).

It should be noted that mice s.c. injected with ADF had a uniform hairline, and the hair length was equal. Hair growth was not uniform in mice treated with e.c. ADF applications (Figure S1).

We found no significant difference regarding the number of HF in BALB/c mice treated with ADF or PBS. This may be explained by the anagen phase of hair growth being prevalent in all skin samples collected at this time point.

Nude mice have a mutation in the transcription factor Foxn1 (nu), resulting in downregulation of hair keratins. FOXN1 deficiency leads to thymic aplasia, alopecia, and nail dystrophy, accounting for the nude/severe combined immunodeficiency (nu/SCID) phenotype in humans and mice [53]. Mice nu/nu were used, for example, to evaluate the efficiency of hair restoration by correcting the follicular keratinization defect, as well as to induce HF regeneration by regulating the Wnt, TGF-β, and BMP signaling pathways [54,55]. In our opinion, these mice may be a model of alopecia caused by chemotherapy or radiation therapy, when not only a defect in the keratinization process is observed, but also suppression of the immune system functioning as a whole. In this case, patients usually experience total hair loss. Nu/nu mice lose all external hair in their first hair cycle after birth, so all mice were at the same phase of the hair growth cycle. This line is suitable for our research because it provides a model in which changes in hair growth and follicle number can be easily assessed.

**Figure 1:**
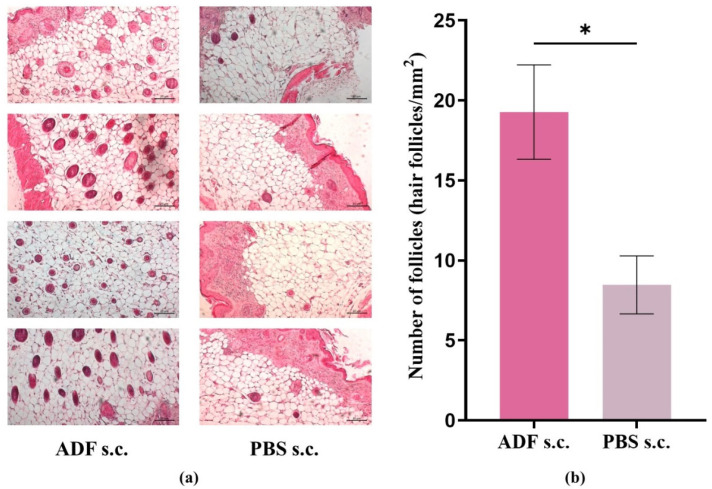
ADF stimulates hair growth. (**a**) Histologic features of ADF- and PBS-treated skin sites in nu/nu mice. (**b**) Quantity of active HF (hair follicles/mm^2^) on the histological skin section. Nu/nu mice were injected with ADF s.c. (2 μg/animal per injection (in a PBS volume of 100 μL)) (“ADF s.c.”) and PBS s.c. (at the same volume (100 μL)) (“PBS s.c.”) on days 1, 3, 5, 7, 9, and 11 (on day 14, skin samples were collected for analysis). Skin sections were stained with hematoxylin-eosin staining and examined at ×100. Scale bar: 20 μm. Mean ± SEM, *—*p* < 0.05.

Thus, to evaluate in detail the ability of ADF to stimulate hair growth, we selected nude/nu mice as a model of alopecia. It was shown that the hair growth was observed in nu/nu mice at the site of ADF administration as well. Histological evaluation was performed to examine the degree of HF activation in nu/nu mice. Hair length was approximately 1 mm as recorded on day 14. In the cross-sections shown, the number of active HF (in the anagen phase) was significantly increased in the ADF-treated group compared to the control group (Figure 1a).

Evaluation of the HF number per mm^2^, presented in Figure 1b, revealed a significant difference between the experimental and control groups (*p* < 0.05).

Thus, the results of histological analysis confirmed the visual assessment data and indicate that ADF enhances hair growth from existing HF.

### 2.2. The Effect of Stimulating Hair Growth Persists After Discontinuation of ADF Administration

**Figure 2:**
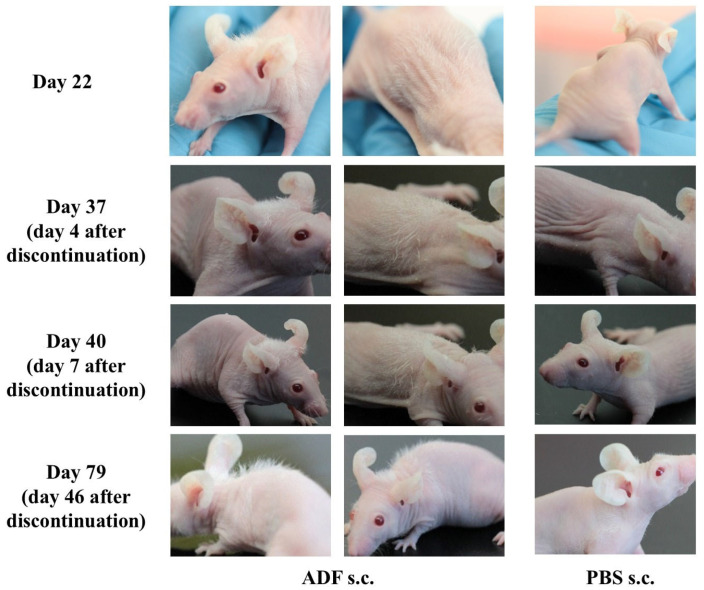
Maintained effect of ADF after discontinuation of injections. The nu/nu mice were injected with ADF s.c. (2 µg/animal per injection (in a PBS volume of 100 μL)) (“ADF s.c.”), and with PBS (in the same volume (100 μL)) s.c. (“PBS s.c.”) every two days for up to 33 days. The nu/nu mice were photographed on day 22, day 37 (4 days after the last ADF administration), day 40 (7 days after the last ADF administration), and day 79 (46 days after the last ADF administration).

Hair growth in nu/nu mice (Figure S2) was recorded as early as day 14 after the first ADF administration. The hair shaft length was approximately 0.5–1 mm on day 14, and 1–2 mm on day 22 of ADF injection. The ADF injections were continued until the day 33 of the experiment. The duration of the effect was then assessed. Hair growth was pronounced on day 37 of the experiment (four days after the last ADF administration) mainly in the injection area. Hair shaft length was 2–3 mm on day 40 (Figure 2).

On day 79, hair growth was observed mainly in the area of ADF injection. The hair shaft length on day 79 was 3–4 mm and the thickness of the visible hair was normal (not vellus hair). Thus, it was shown that ADF stimulates hair growth. It is important to note that this effect persisted even after the discontinuation of ADF injections.

### 2.3. Evaluation of Gene Expression Involved in the Development and Functioning of Hair Follicles

It is known that WNT10B stimulates hair growth [56,57], and that the expression of this gene is stronger in the anagen phase than in the catagen and telogen phases [58]. Leiros et al. showed that *Wnt10b* expression was decreased in patients with alopecia areata and androgenetic alopecia [59].

**Figure 3:**
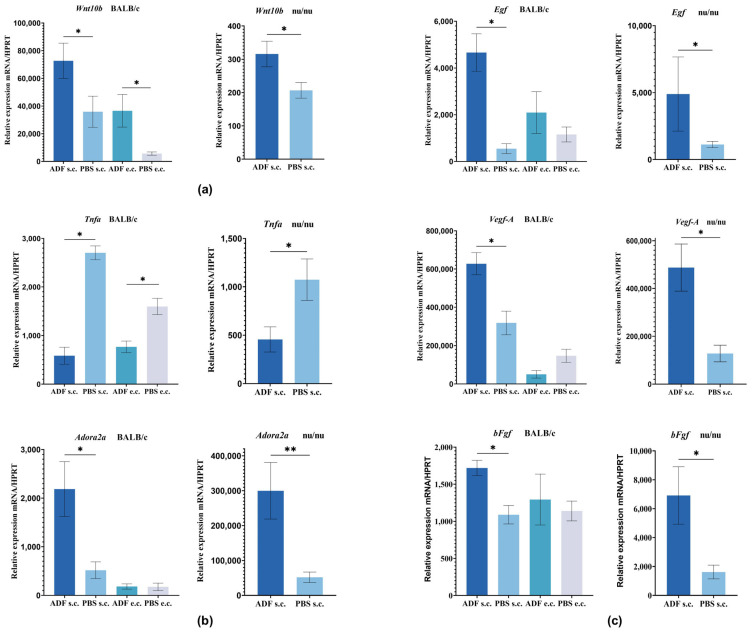
mRNA expression in skin tissue of BALB/c and nu/nu mice. (**a**) The *Wnt10b* mRNA in skin tissue of BALB/c and nu/nu mice; (**b**) The *Tnfα* and *Adora2A* genes expression in skin samples from BALB/c and nu/nu mice; (**c**) The angiogenetic factors (*EGF, VEGF-A, bFGF*) gene expression in skin samples from BALB/c and nu/nu mice. “ADF s.c.”—mice treated with ADF s.c., “PBS s.c.”—mice treated with PBS s.c., “ADF e.c.”—mice treated with ADF e.c., “PBS e.c.”—mice treated with PBS e.c.; Mean ± SEM, *—*p* < 0.05, **—*p* < 0.01.

Gene expression was analyzed by qRT-PCR. Analysis of the *Wnt10b* mRNA level in skin samples of BALB/c mice showed a significantly increased expression in mice treated with ADF s.c. or e.c. compared to the control PBS group (Figure 3a). It should be noted that *Wnt10b* expression was higher in the group subjected to s.c. administration compared to e.c. applications.

Analysis of the *Wnt10b* mRNA level in nu/nu mice also showed significant increase in expression (1.5 times higher compared with group “PBS”) after ADF s.c. injection (Figure 3a). The obtained data indicated that in nu/nu mice treated with ADF, *Wnt10b* gene expression was increased in groups with both s.c. and e.c. administration. This fact may indicate the initiation of the Wnt signaling pathway. Increased *Wnt10b* expression promotes the acceleration of the transition from the telogen phase to the anagen phase in the hair growth cycle and results in the stimulation of hair growth.

In the case of alopecia, there is a deviation from the normal distribution of growth factors in the HF structures and microenvironment. Data strongly suggest that EGF and the FGF family play important roles in the processes of HF morphogenesis. Factors VEGF-A and EGF are two growth factors that are strongly associated with the angiogenesis process. EGF stimulates the proliferation and migration of keratinocytes. Studies by Mak and Chan have shown that continuous expression of EGF prevented HF from entering the catagen phase and that it is involved in the new circulatory network formation around HF (the anagen phase) [60]. The mechanisms underlying the transition from phase to phase remain largely unknown.

It is known, that M2 macrophages produce various growth factors, including VEGF-A, EGF, and bFGF, which are critical for the regulation of the hair cycle and hair growth. In vitro studies have revealed even the possibility of synergy between VEGF and bFGF in the induction of angiogenesis [61]. We demonstrated that the levels of Vegfa, bFGF, and Egf expression significantly increased in mice treated with ADF s.c. (Figure 3c). This data indicate that ADF has the ability to activate angiogenesis in the area of HF development, activating HF morphogenesis and promoting hair growth in BALB/c and nu/nu mice. It is possible that this effect is related to the ability of ADF to stimulate macrophage activity. We have tried to substantiate this assumption below.

Another factor involved in the pathogenesis of various alopecia types is TNFα. Recent studies have shown that TNFα plays a central role in growth inhibition and apoptosis of HF and also coordinates the hair growth cycle in the alopecia pathogenesis [62]. In vivo studies in TNFα-deficient mice have shown a delay in the onset of the catagen phase, and it has been suggested that TNFα induces apoptosis at the onset of catagen [63,64,65,66]. Shah et al. showed that anti-TNFα therapy slowed down hair loss in patients with inflammatory diseases, but the mechanisms of this effect are unclear [67]. The TNFα inhibitor (adalimumab), which effectively treats cases of alopecia universalis, has demonstrated that negative regulation of TNFα is effective in the treatment of alopecia areata [68].

Figure 3b shows the *Tnfα* gene expression in skin samples from BALB/c and nu/nu mice. *Tnfα* expression was significantly reduced in BALB/c mice treated with ADF relative to both PBS e.c. and PBS s.c. groups. In addition, we observed a trend toward decreased *Tnfα* expression in nu/nu mice treated with ADF. Apparently, ADF is able to reduce the potential negative effects of TNFα on HF.

It is known that suppression of TNFα can occur through activation of the adenosine A_2A_ receptor, also known as ADORA2A (ligand is adenosine) [17]. In our study, the levels of *Adora2A* expression were increased in BALB/c and nu/nu mice treated with ADF s.c. (Figure 3b).

It is known that the mechanism of minoxidil action and its effect on hair growth stimulation is also mediated by the adenosine receptor. Minoxidil was shown to increase intracellular calcium ([Ca^+2^]i) levels and VEGF-A production in dermal papillary cells (DPCs) in a manner similar to adenosine, and this increase was inhibited by an adenosine receptor antagonist. Increased [Ca^+2^]i and VEGF-A production in turn stimulated the DPC proliferation. It is assumed that the sulfonylurea receptor (SUR) is the target receptor for minoxidil and the source of ATP and adenosine production, respectively [69].

Structurally, adenosine receptors penetrate the cell membrane in seven “stitches.” Inside the cell, they also form a loop to which G protein is attached. The ligand, which is a chemical signal, enters the binding site from the outside. The gap between the “stitches” of the receptor—which changes the conformation of the receptor (including from the inside) and switches the G protein to a different position. It triggers a cascade of biochemical changes inside the cell, ultimately leading to various responses. ADORA2A activation causes a wide range of responses that can be broadly classified as anti-inflammatory [70]. In response to stress or injury, adenosine has a predominantly cytoprotective effect. It protects tissues from damage in cases of hypoxia, ischemia or convulsions, promoting the production of vascular endothelial growth factor by mononuclear phagocytes. It is described in the literature that a derivative of fullerene C60 is able to stimulate the expression of this receptor in a human neuroepithelioma cell line [71]. Based on the data obtained, the authors suggested a neuroprotective effect of the compound.

Previously, we received experimental data regarding the biological activity of ADF, including the presence of anti-inflammatory, anti-allergic and wound-healing effects [32,36], which are similar to those caused by activation of the adenosine receptor. In addition, data were obtained regarding the ability of ADF to influence the expression of the adenosine receptor gene, as well as the associated Tnfα. These premises formed the basis for our assumption that the mechanism of ADF action may be mediated by activation of the ADORA2A.

### 2.4. Hypothesis About Possibility of Interaction of Fullerene C60 with Adenosine A2A Receptor

**Figure 4:**
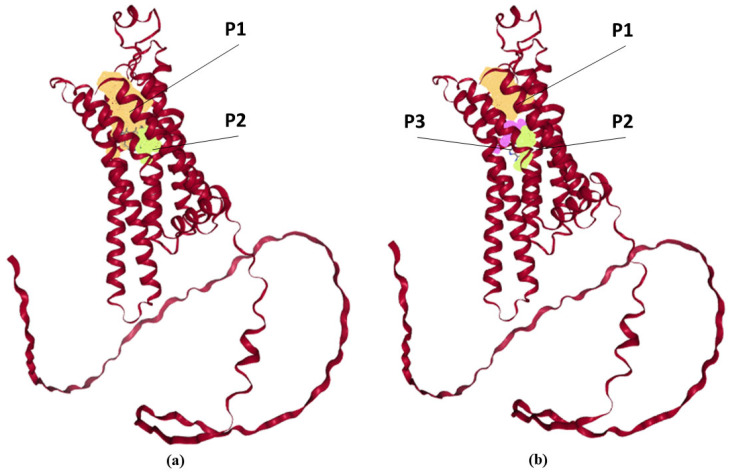
Predicted ligand-binding “pockets” with ADORA2A obtained from Proteins Plus («https://proteins.plus/» (accessed on 1.03.2025)) were processed by DoGSiteScorer3 (binding site detection). (**a**) Two predicted ligand-binding “pockets” of the ADORA2A for the fullerene C60 (orange and light green grid representations of pockets “P1” and “P2”). (**b**) Three predicted ligand-binding “pockets” of the ADORA2A for adenosine (orange, light green, and pink grid representations of pockets “P1”, “P2”, and “P3”).

It is known that ADORA2A activation requires the formation of two hydrogen bonds, hydrophobic connections, and aromatic interactions. The fullerene C60 molecule structure contains a large number of aromatic rings and it is hydrophobic, indicating the possibility of C60 interaction with ADORA2A. Thus, in our opinion, due to the hydrophobicity of the C60 fullerene molecules themselves, ADF may be capable of carrying out aromatic interactions and forming hydrophobic bonds. We therefore analyzed the probability of a relationship between fullerene C60 and ADORA2A. A grid representation of the pockets is displayed in Figure 4.

Collected data for ligand-binding “pockets” of the ADORA2A is presented in Table 1. The adenosine molecule (adenosine receptor agonist) was chosen for comparison. The figure shows that the pockets obtained for the probable interaction of the receptor with the ligand are located inside the receptor. Moreover, the calculated size of the binding cavities fully allows receptor interaction with the C60 fullerene molecule. Probably, the fullerene C60 molecule can interact with amino acid (AA) residues within these cavities. The size of the ADORA2A cavity, where the active center of the receptor is located, is 9.5–12.82 Å. It was shown that adenosine (ligand for adenosine receptor) molecule size is about 10 Å (length) and 6 Å (width), while fullerene C60 is a sphere with a diameter of 7.2 Å, which makes the surface area of the two molecules comparable. Table 1 shows that the amino acid residues located near the ligand-binding pockets and calculated for adenosine and C60 fullerene are almost completely identical. The residues located close to the ligand-binding pockets are mostly nonpolar (Ala, Leu, Val), polar (Ser, Thr) and aromatic (Phe, Tyr), thereby implying that the protein–ligand binding may be stabilized by hydrophobic interactions. The fullerene C60 binding site may contain hydrophobic cavities rich in Trp, Phe, and Tyr groups, and the π-system of fullerene C60 may interact with these residues.

Therefore, the molecular modeling indicates the possibility of an interaction between fullerene C60 and ADORA2A.

It was shown, in silico, that the fullerene C60 molecule may bind to ADORA2A due to the fact that pockets for possible fullerene C60 interaction are located identically to the pockets for the adenosine. We assume that the fullerene C60 molecule is capable of changing the ADORA2A conformation similar to adenosine, which may influence ADORA2A-associated signaling.

### 2.5. The Proposed Mechanism for Stimulating Hair Growth by Fullerene C60

**Figure 5:**
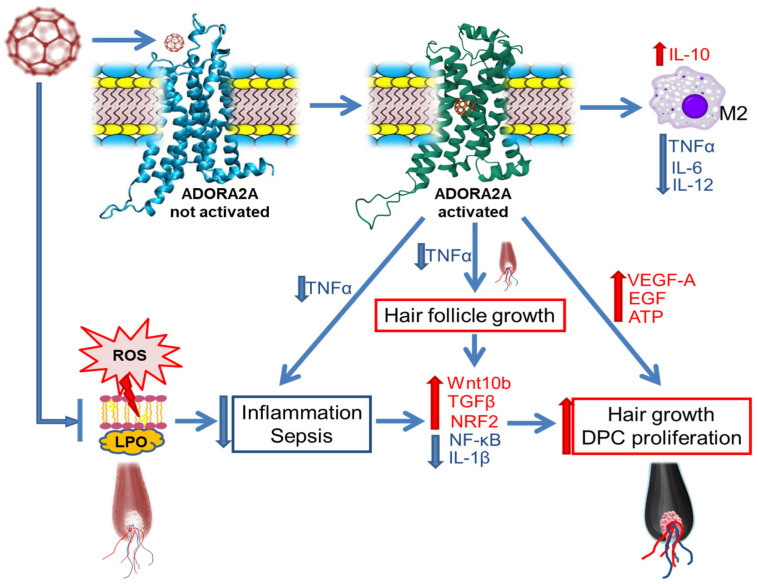
Scheme of the proposed mechanism of the fullerene C60 biological activity of stimulating hair growth and reducing inflammation. Inscriptions in blue indicate a decrease in the indicator; inscriptions in red indicate an increase in the indicator. ADORA2A—adenosine A2A receptor; ROS—reactive oxygen species; DPC—dermal papilla cells; LPO—lipid peroxidation; M2—macrophages of M2 phenotype; TNFα—tumor necrosis factor; ATP—adenosine triphosphate; TGFβ—transforming growth factor beta; IL-10—interleukin-10; NRF2—nuclear factor erythroid 2-related factor 2; NF-κB—nuclear factor kappa-light-chain-enhancer of activated B cells; IL-1β—interleukin-1β; EGF—epidermal growth factor; VEGF-A—vascular endothelial growth factor alpha.

To visualize the proposed mechanism of hair growth stimulation by fullerene C60 the following scheme is proposed (Figure 5).

The diagram visualizes that ADORA2A activation leads to *Tnfα* expression inhibition, which mediates stimulation of HF growth. Activation of this receptor stimulates the expression of angiogenesis factors *Vegfa* and *Egf*, which also promote the growth and development of HF, and stimulate dermal papilla cells proliferation. At the same time, the Wnt signaling pathway, which regulates the growth and development of hair follicles, is activated. Inhibition of the *Tnfα* expression leads to a reduction of inflammation in the alopecia area and activation of the cell’s antioxidant system, which is accompanied by stimulation of the antioxidant defense factor *Nrf2* production. We assume that reduction of the inflammation can also occur due to the interaction of fullerene C60 molecules with cell membrane phospholipids, blocking the process of lipid peroxidation, which in some cases can be the cause of hair loss.

The scheme shows that ADORA2A activation may also influence the differentiation process of macrophages toward M2. Macrophages express adenosine receptors, which can dictate classical macrophage activation. For example, adenosine has been shown to inhibit the release of TNFα, IL-6 and IL-12 and to increase the production of IL-10 and VEGF-A [72] and may also enhance the IL-4 and IL-13-induced alternative activation of peritoneal macrophages through a TLR4-independent mechanism [73].

Previously, we demonstrated the possibility of this kind of interaction in vitro. In particular, the ability of ADF to influence the differentiation of macrophages was revealed. Thus, in the presence of ADF, differentiation of M0 macrophages predominantly led to the appearance of the M2 phenotype [74]. At the same time, the expression level of *Nrf2* was increased, but the production of the pro-inflammatory factors *Tnfa*, *Il-6*, *Il-1b*, and *Nfkb* was reduced.

Thus, we suggest that in addition to ADORA2A (one of the C60 targets), the hair growth effect, as well as the anti-inflammatory action of ADF, may be due to the C60 interaction with macrophages.

### 2.6. Hypothesis About the Interaction of ADF with Macrophages

**Figure 6:**
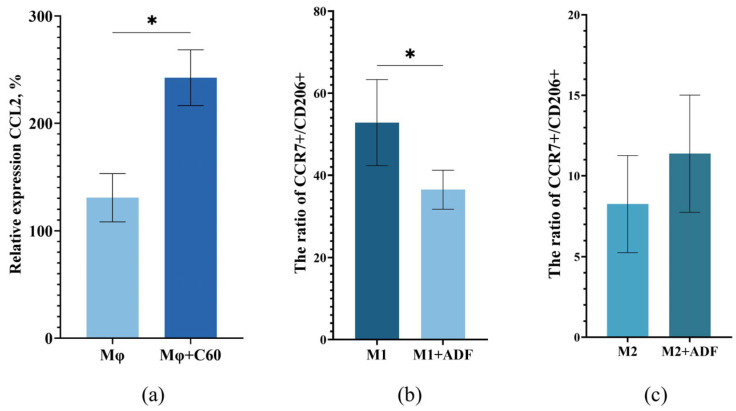
(**a**) The level of *Ccl2* expression. Mφ—macrophages were incubated without ADF; Mφ+C60—macrophages were incubated in the presence of ADF. The data are shown as mean ± SD (*n* = 6). (**b**,**c**) Effect of ADF on macrophage polarization. Data are presented as the ratio of CCR7+ to CD206+. M1—Mφ with added LPS and IFNγ, M2—Mφ with added IL-4 and IL-13. Data are shown as mean ± SD (*n* = 4). *—*p* < 0.05.

One of the most potent monocyte/macrophage chemokines is chemokine CCL2 [75]. CCL2 is required to stimulate macrophage recruitment to various sites of injury and infection. CCL2 cytokine release in the hair follicle induces macrophage infiltration in the hair microenvironment. Fullerene C60 is a nanoparticle, so, theoretically, it can attract macrophages. We analyzed the ability of ADF to influence chemotaxis and differentiation of monocytes/macrophages. To assess the influence of ADF on the functioning of Mφ, the chemokine CCL2 expression was analyzed (Figure 6a).

It was shown that in the presence of ADF, cells expressed 1.8 times the chemotaxis factor *Ccl2*, which apparently indicated the ability of fullerene C60 to significantly enhance macrophage chemotaxis. Thus, it can be assumed that the effect of hair growth activation in mice of different lines treated with ADF, which we demonstrated, may be associated with the attraction of macrophages to the hair follicle area. However, it has been previously shown that macrophages are widely distributed around the HF and most of them were polarized to the M2 type in anagen. Apparently, this is due to the fact that a number of factors which can be expressed predominantly by M2 macrophages are necessary for hair growth activation. M2-type macrophages are involved in the secretion of growth factors and angiogenesis—VEGF-A and EGF, promoting cell proliferation and tissue development [76]. It was also found that CD206+ M2 macrophages produced growth factors important in Wnt-pathway signaling, including bFgf, and activate the Wnt/β-catenin signaling pathway [77,78]. At the next stage of the study, it was interesting to determine the ability of ADF itself to influence the differentiation of macrophages. For this purpose, macrophages were obtained using human PBMCs. Macrophages treated with LPS and IFN-γ acquired the M1 phenotype (as evidenced by the M1 marker CCR7), while macrophages treated with IL-4 and IL-13 acquired the M2 phenotype (as evidenced by the M2 marker CD206). After preliminary incubation of cells with ADF the phenotype of the obtained cells was characterized by flow cytometry. The number of cells expressing CCR7 as a marker of M1 and CD206 as a marker of M2 were determined. For clarity, the results are presented as a ratio of the number of cells expressing CCR7 to cells expressing CD206 (CCR7+/CD206+) (Figure 6b,c). It was shown that the expression of these markers changes on cells treated with ADF. Thus, preliminary incubation of M1 cells with ADF led to a statistically significant reduction in the number of CCR7+ compared to cells without ADF treatment (Figure 6b). It is important to note that incubation of M2 cells with ADF did not affect the number of CD206+ cells (Figure 6c). To visualize the results, we presented photos of the macrophages that we observed under the microscope. The photos show that the phenotype of the macrophages treated or non-treated with ADF is different. It is clear that in the well where the cells were stimulated with IFNγ and LPS, the cells acquired the M1 phenotype (elongated cells), and after stimulation with IL-4 and IL-13—the M2 phenotype (rounded cells). It should be noted that with preliminary macrophage incubation with ADF and subsequent stimulation with IFNγ and LPS, the macrophages acquire a phenotype that is similar to M1, but only partially. A very significant number of cells acquire a rounded shape, similar to M2. When M0 are stimulated with IL-4 and IL-13 after preliminary incubation with ADF, macrophages with the M1 phenotype are absent and their phenotype is similar to M2 (Figure S3). We also showed that undifferentiated macrophages treated with ADF alone had reduced expression levels of M1-specific cytokines (TNFα, IL-6) and increased expression levels of M2-specific factors (TGFb, IL-10, VEGF-A). The presented data may indicate the ability of ADF to some extent to initiate a shift in macrophage differentiation toward M2, which provides in the HF microenvironment a favorable basis for activation hair follicle cell proliferation and promote the development of a new anagen [20]. This assumption is consistent with the presence of local anti-inflammatory activity in ADF—previously demonstrated by our group [32]—which may also be due to the macrophages attraction to the inflammation area and a shift in the Mφ differentiation toward M2. It is important to note that M2 polarization of macrophages may also depend on the presence of low levels of reactive oxygen species (ROS). ROS are essential for hair follicle generation by promoting HF cell proliferation [79]. At the same time, an increase in ROS is a key factor in HF degeneration during catagen [80]. More and more data support that ROS clearance (the process of removing harmful ROS that depends on the action of antioxidant agents) promotes HF regeneration [81]. Fullerene C60 and its derivatives are powerful antioxidants and are capable of inactivating ROS and free radicals in vivo and in vitro. Based on the above data, it can be assumed that M2 macrophages attracted by ADF are involved in the activation of HF. Macrophages release growth factors near the HF, including EGF, bFGF, VEGF-A, which can initiate and maintain the anagen phase. Thus, targeting the HF microenvironment with ADF can promote HF activation and hair growth.

Currently, data on the toxicity of fullerenes are numerous but contradictory. The ambiguity of research results complicates the transition from scientific research to the development of real drugs. The ability of fullerenes to accumulate in some organs and tissues is in most cases interpreted as their disadvantage, while a number of studies have shown that there is no connection between the accumulation of fullerenes and toxic effects. The pharmacokinetics and toxicity of fullerenes depend on the method of obtaining the fullerene, the route of its administration, and are also largely associated with their functionalization, since pure fullerenes are usually harmless. These factors must be taken into account when developing new drugs based on fullerenes [82].

It should be noted that, in earlier work, ADF biocompatibility and absence of toxicity were showed in experiments in vivo and in vitro [32,52]. The results of our earlier in vitro tests showed absence of cytotoxicity in ADF in A549, HepG2, and HeLa cell lines, confirming high biocompatibility of ADF. The histological analysis of the internal organs and tissues of experimental mice in acute and chronic toxicity studies showed the absence of toxic and irritating effects of ADF at different routes of administration [32].

## 3. Materials and Methods

### 3.1. Mice

Female 4–6-week-old BALB/c mice (Stolbovaya, Moscow, Russia) and 6–8-week-old nu/nu female mice (Puschino, Russia) were kept under pathogen-free conditions. Experiments with animals were carried out in accordance with the EU Directive 2010/63/EU for animal experiments. All experimental protocols were reviewed and approved by the local ethical committee of NRC Institute of Immunology FMBA of Russia (protocol No.7 from 21 September 2020). All mice were provided with official documentation, including a certificate of health and a certificate of genetic background purity. Each group of animals participating in the experiment was kept in individual cages of 5–6 mice (to minimize potential confounders). The following conditions were maintained in the vivarium: air temperature 18–26 °C; relative humidity 30–70%. All mice had unlimited access to drinking water and food.

The experiments involved the minimum required number of animals to obtain statistically significant results (*p* values < 0.05). Sample size was determined in accordance with institutional ethical guidelines for laboratory animal research. The criteria used for inclusion were age (4–6-week-old for BALB/c; 6–8-week-old for nu/nu) and sex (female mice). All animals participated in the experiment until its completion, and the material from all animals was analyzed. Animals were randomly assigned to experimental groups using a computer-generated random number sequence to reduce bias in animal selection and outcome assessment.

### 3.2. The Aqueous Dispersion of Fullerene C60 (ADF)

ADF was obtained by method described earlier [83]. Briefly, 20 mg of crystalline C60 99.9% (SES Research, Houston, TX, USA) were dissolved in 25 mL of N-methylpyrrolidone 99% (Panreac, Barcelona, Spain) on magnetic stirrer and resulting dark-brown solution was mixed with 100 mL deionized water. The obtained solution was stirred for 1 h and then subjected to exhaustive dialysis (dialysis tubes 6–8 kDa Spectra/Por (Spectrum labs, Piraeus, Greece)) against deionized water. The final dialysis solution was filtered through a 0.45 mm nitrocellulose membrane, resulting in a clear, transparent solution with a brownish-yellow color and a concentration of 120 µg/mL and good stability. This biocompatible method of preparing ADF uses non-toxic solvents, ultrasonic treatment, and heating, making it suitable for further application in medicine.

### 3.3. Experimental Protocol

The backs of female BALB/c mice (six animals per group, 24 animals for experiment) were shaved and epicutaneous (e.c.) ADF applications (at a dose of 20 μg fullerene C60/animal in 200 µL PBS) or PBS alone (200 µL) were applied topically on the mouse each day for up to day 15. In addition, the ADF (2 µg/animal) or PBS (200 µL) were subcutaneously (s.c.) injected for each day for up to day 15. S.c. injections and cutaneous applications were applied on day 1, 3, 5, 7, 9, 11, 13, and 15. On day 16, skin samples were collected for quantitative real-time PCR (qRT-PCR) and histological examination.

An experimental group of nu/nu mice (five animals per group, 10 animals for experiment) was s.c. injected with ADF (2 µg/animal) in PBS (100 μL). Control animals received PBS at the same volume. Subcutaneous injections were carried out on day 1, 3, 5, 7, 9, and 11. On day 14, skin samples were collected from the ADF/PBS injection site for qRT-PCR and histological examination. Analyses were carried out as described in the Section 3.4, Section 3.5 and Section 3.6.

When analyzing the effect of hair growth stimulation with long-term administration of ADF, nu/nu mice (five mice per group, 10 mice for experiment) were treated with ADF via s.c. injection (2 μg/animal in 100 μL PBS), and the control group of animals was administered with PBS alone at the same volume. S.c. injections were carried out each day for up to day 33. The experiment was terminated on day 79. Throughout the experiment mice were photographed (on days 22, 37, 40, and 79).

The indicated doses of ADF were selected empirically based on the visual effect. The dose of ADF for epicutaneous application is 10 times greater than for subcutaneous application due to lower bioavailability.

### 3.4. Skin Biopsy for Histological Analysis and qRT-PCR

Skin biopsy samples were collected on days 16 and 14 from BALB/c and nu/nu, respectively. Samples were fixed with 10% paraformaldehyde and embedded in paraffin for histological assay. Skin biopsy samples were treated with Trizol overnight, followed by homogenization, mRNA extraction, and qRT-PCR analysis.

### 3.5. Quantitative Real-Time PCR

The total RNA from mouse skin biopsies was extracted by using the RNeasy Mini Kit (Qiagen) according to the manufacturer’s instructions and thereafter reverse-transcribed into cDNA using Reverta-L kit (Interlabservice, Moscow, Russia). The reverse transcription reaction product was amplified by qRT-PCR using an iCycleriQ Real-time PCR Detection System (Bio-Rad Laboratories, Hercules, CA, USA). Relative quantification of qRT-PCR was used to detect changes in expression of the target genes relative to a reference gene, which is the housekeeping gene HPRT. The results are presented as arbitrary units.

### 3.6. Histological Analysis and Counting of the Number of Hair Follicles

Skin tissue was treated with alcohols of increasing concentration and in the alcohol-chloroform, chloroform-paraffin and paraffin system, then embedded in paraffin (SLEE Mainz, Nieder-Olm, Germany). Fixed skin tissue samples (section thickness—4 microns) (Finesse E+, Cambridge, UK) were stained with hematoxylin and eosin (H&E). The histological alteration was analyzed under a microscope Zeiss Axio Imager A1, Oberkochen, Germany at 100×—fold magnification. The number of HF was evaluated in eight fields of vision in a blinded manner.

Hair follicles were counted using an OMP N632639, Tokyo, Japan object micrometer. For this purpose, the diameter of the lens field of view (N PLAN, 20×/0.40) was determined to be 1 mm. Based on the formula for determining the area of a circle S = π·r^2^, where π = 3.14, r—the radius of the circle, the area of one field of view was calculated, which was equal to 0.8 mm^2^. Next, the number of HF of all growth phases was counted in 10 fields of view of the microscope using the above objective. The obtained result was expressed as the number of follicles per 8 mm^2^, which was then normalized to 1 mm^2^.

### 3.7. Computational Modeling of Protein Structures

Determination of interaction probability between fullerene C60 and ADORA2A were performed in silico, using visualization and molecular modeling methods.

Structural data of the mouse adenosine A_2A_ receptor protein (UniProt Q60613) [84] was retrieved from AlphaFold Protein Structure Database (https://www.alphafold.com/ (accessed on 1 March 2025)) [85,86]. The structure of fullerene C60 (compound ligand) was downloaded from RCSB Protein Data Bank (RCSB.org) [87].

The protein interaction was visualized using the Structure-Based Modeling Support Server (https://proteins.plus/ (accessed on 1 March 2025)) [88,89,90]. Binding sites and potential binding pockets were predicted using the program DoGSite3—a grid-based method which uses a Difference of Gaussian filter to detect potential binding pockets. Properties, describing the size, shape and chemical features of the predicted pockets are calculated [91].

### 3.8. Macrophage Chemotaxis Assay

Mouse peritoneal macrophages were obtained by lavaging the peritoneal cavity with phosphate-buffered saline. Mice were euthanized, the peritoneum was aseptically opened, and the peritoneal cavity was lavaged with 5 mL of PBS using tweezers and a pipette. To prevent cell adhesion to the plastic, the tube with the peritoneal cell suspension was kept in the ice bath. The suspension was centrifuged at 250× *g* for 10 min, then the supernatant was removed and a complete nutrient medium based on DMEM was added. Next, the cells were seeded in a 6-well plate (100–150 thousand cells/well), where a drop of ADF was previously added. The polymerase chain reaction with real-time detection was chosen to determine the level of expression of the chemokine (C-C motif) ligand 2 (CCL2).

### 3.9. Isolation of Peripheral Blood Mononuclear Cells

Human peripheral blood mononuclear cells (PBMCs) were isolated by sedimentation in a Ficoll-400 density gradient according to the standard protocol (STEMCELL Technologies, Vancouver, BC, Canada) and seeded in a 6-well culture plate at a concentration of 20 million cells/well. Then, the cells were incubated overnight at 37 °C, 5% CO_2_ in IMDM culture medium (Bioinnlabs, Moscow, Russia) supplemented with 10% FBS (HyClone, Marlborough, MA, USA), sodium pyruvate (100×) (Gibco, New York, NY, USA), hepes (100×) (Capricorn, Dusseldorf, Germany), insulin (500×) (PanEco, Moscow, Russia), and glutamax (Gibco, New York, NY, USA) (100×).

Protocols for clinical biospecimen collection from volunteers were reviewed and approved by local ethical committee of NRC Institute of Immunology FMBA of Russia (protocol No.4, 16 May 2023). All volunteers provided written informed consent before biospecimens were collected.

### 3.10. Differentiation of PBMCs

To obtain Mφ macrophages from PBMCs, non-adherent cells were removed 24 h after seeding and the IMDM medium was replaced with IMDM+M-CSF (5 μg/mL) (SCI-STORE, Moscow, Russia) in a volume of 2 mL/well. The cells were incubated for 4 days at 37 °C and 5% CO_2_. Then, another half of the IMDM+M-CSF (5 μg/mL) from the initial volume of the medium was added. After 24 h of incubation at 37 °C and 5% CO_2_, ADF (10 μg/mL) was added. Twenty-four hours after cell incubation at 37 °C and 5% CO_2_, the medium with ADF was removed and the medium from the “donor wells”, in which the cells were incubated under the same conditions but without the addition of ADF, was added. Next, LPS (Lipopolysaccharides from Vibrio cholerae Inaba 569B, LPS, Sigma, St. Louis, MO, USA) (10 ng/mL) and IFNγ (ABclonal, Woburn, MA, USA) (50 ng/mL) were added to the wells to polarize macrophages to the M1 phenotype or IL-4 (10 ng/mL) and IL-13 (20 ng/mL) (ABclonal, Woburn, MA, USA) to polarize macrophages to the M2 phenotype. Then, 48 h after incubation at 37 °C and 5% CO_2_, the cells were lysed using lysis buffer (Qiagen, Hilden, Germany) containing 1% β-mercaptoethanol and RNA was isolated (Qiagen, Hilden, Germany) according to the manufacturer’s instructions.

### 3.11. Flow Cytometry

Macrophages phenotyping was performed by visual analysis of cells under a microscope, as well as by flow cytometry according to a standard protocol. Antibodies to CD206 and CCR7 were used for the analysis.

To assess the surface expression of macrophage markers, tissue samples were stained for flow cytometry with a fluorochrome-conjugated monoclonal antibody against CCR7 to identify M1, while a fluorochrome-conjugated monoclonal antibody against CD206 will identify M2.

### 3.12. Statistical Analysis

Prior to statistical analysis, all data were tested for compliance with the assumptions of parametric tests. Normality of distribution was assessed using the Shapiro–Wilk test. Data were presented as the means ± SE. All statistical analyses were performed with GraphPad Prism 9 software. The significance of the results was determined by using a Mann-Whitney test and one-way ANOVA test (Kruskal–Wallis test), *p* values < 0.05 were considered to be statistically significant.

## 4. Conclusions

Our study suggests that ADF attracts macrophages into the microenvironment of HF and promotes their polarization to M2 phenotype. M2 macrophages are capable of secreting growth and angiogenesis factors that play a key role in the development and functioning of HF. These factors stimulate the proliferation of hair follicle cells and, by means of synergistic action, could promote initiation of the anagen phase in resting hair follicles and stimulate hair regrowth.

Thus, an increase in the number of macrophages and their differentiation to the M2 phenotype leads to the regeneration of HF. At the same time, the demonstrated increase in the expression of the adenosine receptor may also contribute to the fullerene C60 follicle-stimulating activity, given the suppression of TNFα and stimulation of the expression of angiogenesis factors VEGF-A, EGF and bFGF.

Thus, the presented study demonstrates ADF-mediated hair growth stimulation effect, as well as possible mechanisms of ADF biological activity, which may be due to the interaction of C60 with the adenosine receptor and macrophages. The presented data on the mechanisms of biological activity of fullerene C60 can become the basis for further research in this area.

## Figures and Tables

**Table 1:** The predicted binding pockets and interacting AA residues of ADORA2A with ligands processed by DoGSiteScorer3.

Ligand	Predicted Pockets				
	Name	Size and Shape Descriptors	Element and Functional Group Descriptors	Amino Acid (AA) Composition	AA Residues
Fullerene C60 sphere 7 Å 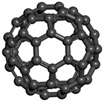	P1 (orange)	Volume 457.216 [Å^3^] Surface 494.781 [Å^2^] Depth 22.127 [Å]	Pocket atoms 195 Hydrogen bond donors 9 Hydrogen bond acceptors 11 Aromatic atoms 39 Hydrophobicity ratio 0.74878	Apolar AA 27 Polar AA 12 Acidic AA 1 Basic AA 3	Tyr 6, Ala 56, Phe 59, Ala 60, Ile 63, Ser 64, Phe 77, Ala 78, Val 81, Leu 82, Val 83, Thr 85, Gln 86, Ile 89, Ser 129, Ile 132, Gly 133, Leu 162, Phe 163, Glu 164, Tyr 171, Met 172, Asn 176, Phe 177, Phe 180, Val 181, Leu 185, Phe 237, Ala 238, Cys 240, Trp 241, Leu 242, Leu 244, His 245, Asn 248, His 259, Pro 262, Met 265, Tyr 266, Ala 268, Ile 269, Ser 272, His 273
	P2 (light green)	Volume 75.776 [Å^3^] Surface 51.825 [Å^2^] Depth 9.499 [Å]	Pocket atoms 64 Hydrogen bond donors 5 Hydrogen bond acceptors 6 Aromatic atoms 12 Hydrophobicity ratio 0.7188	Apolar AA 8 Polar AA 5 Acidic AA 1 Basic AA 1	Leu 45, Ala 48, Asp 49, Val 52, Ala 56, Val 81, Leu 84, Thr 85, Ser 88, Phe 237, Trp 241, Ser 272, His 273, Asn 275, Ser 276
Adenosine ligand size 10 × 6 Å 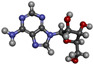	P1 (orange)	Volume 335.36 [Å^3^] Surface 408.501 [Å^2^] Depth 14.333 [Å]	Pocket atoms 92 Hydrogen bond donors 8 Hydrogen bond acceptors 9 Aromatic atoms 21 Hydrophobicity ratio 0.7826	Apolar AA 16 Polar AA 5 Acidic AA 1 Basic AA 2	Tyr 6, Ala 56, Phe 59, Ala 60, Ile 63, Ser 64, Phe 77, Ala 78, Val 81, Leu 82, Leu 162, Phe 163, Glu 164, Met 172, Trp 241, Leu 244, Asn 248, His 259, Pro 262, Met 265, Tyr 266, Ile 269, Ser 272, His 273
	P2 (light green)	Volume 79.36 [Å^3^] Surface 52.698 [Å^2^] Depth 10.911 [Å]	Pocket atoms 69 Hydrogen bond donors 4 Hydrogen bond acceptors 6 Aromatic atoms 11 Hydrophobicity ratio 0.7101	Apolar AA 9 Polar AA 6 Acidic AA 1 Basic AA 1	Leu 45, Ala 48, Asp 49, Val 52, Val 81, Leu 84, Thr 85, Ser 88, Leu 92, Ile 233, Phe 237, Trp 241, Ser 272, His 273, Asn 275, Ser 276, Asn 279
	P3 (pink)	Volume 61.44 [Å^3^] Surface 32.276 [Å^2^] Depth 12.825 [Å]	Pocket atoms 81 Hydrogen bond donors 1 Hydrogen bond acceptors 2 Aromatic atoms 17 Hydrophobicity ratio 0.7407	Apolar AA 12 Polar AA 6 Acidic AA 0 Basic AA 1	Leu 82, Val 83, Thr 85, Gln 86, Ile 89, Ser 129, Ile 132, Gly 133, Tyr 171, Met 172, Asn 176, Phe 180, Val 181, Leu 185, Phe 237, Ala 238, Trp 240, Leu 242, His 245

## Data Availability

No datasets were generated or analyzed during this study. Data will be made available on request.

## Supplementary Materials

The following supporting information can be downloaded at: https://www.mdpi.com/article/10.3390/ijms26178517/s1.

## Abbreviations

The following abbreviations are used in this manuscript:

ADF	Aqueous dispersion of unmodified fullerene C60
PBS	Phosphate-buffered saline
HF	Hair follicle
VEGF-A	Vascular endothelial growth factor alpha
EGF	Epidermal growth factor
bFGF	Basic fibroblast growth factor
ADORA2A	Adenosine A2A receptor
ROS	Reactive oxygen species
DPC	Dermal papilla cells
LPO	Lipid peroxidation
Mφ	Macrophages
M1	Macrophages of the M1 phenotype
M2	Macrophages of the M2 phenotype
TNFα	Tumor necrosis factor alpha
ATP	Adenosine triphosphate
TGFβ	Transforming growth factor beta
IL-10	Interleukin-10
NRF2	Nuclear factor erythroid 2-related factor 2
NF-κB	Nuclear interleukin-1β factor kappa-light-chain-enhancer of activated B cells
IL-1β	Interleukin-1β
e.c.	Epicutaneously
s.c.	Subcutaneously
