# Supplementary material for: Aqueous Dispersion of Unmodified Fullerene C60: Stimulation of Hair Growth and Study of a New Molecular Target for Interaction
Source: Int J Mol Sci. 2025 Sep 2;26(17):8517. doi: 10.3390/ijms26178517 (PMC12429516; doi:10.3390/ijms26178517)
Supplement: Supplementary file 1 [file ijms-26-08517-s001.zip › ijms-3794401-SI.pdf]

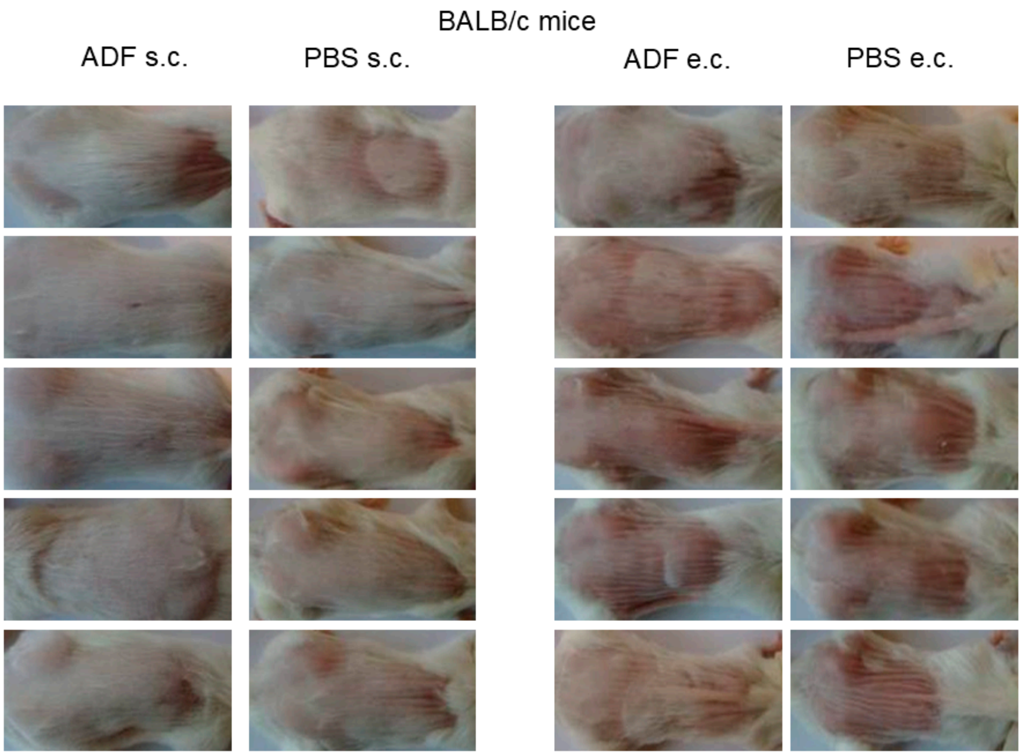

Figure S1. The effect of epicutaneous application and subcutaneous injections of ADF in shaved BALB/c on the hair growth. The back of female BALB/c mice were shaved and epicutaneous (e.c.) ADF applications (at a dose of 20 μg fullerene C60/animal in 200 μl PBS) or PBS alone (200 μl) were applied topically on the mouse every two days for up to day 15. In addition, the ADF (2 μg/animal) or PBS (200 μl) were subcutaneously (s.c.) injected for every two days for up to day 15. S.c. injections and e.c. applications were applied on day 1, 3, 5, 7, 9, 11, 13 and 15. The BALB/c mice were photographed on day 16.

Nu/nu mice before injection of ADF Day 1

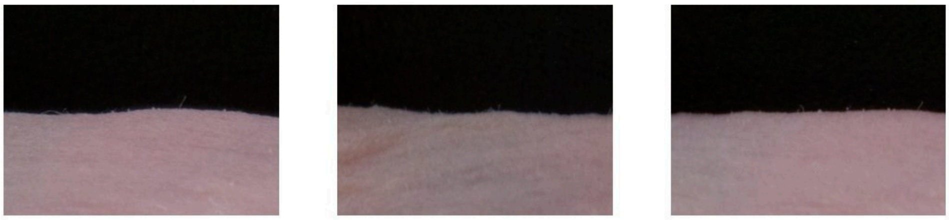

Figure S2. The original image of nu/nu mice before injection of ADF. Close-up view of animal's skin on day 1 of experiment.

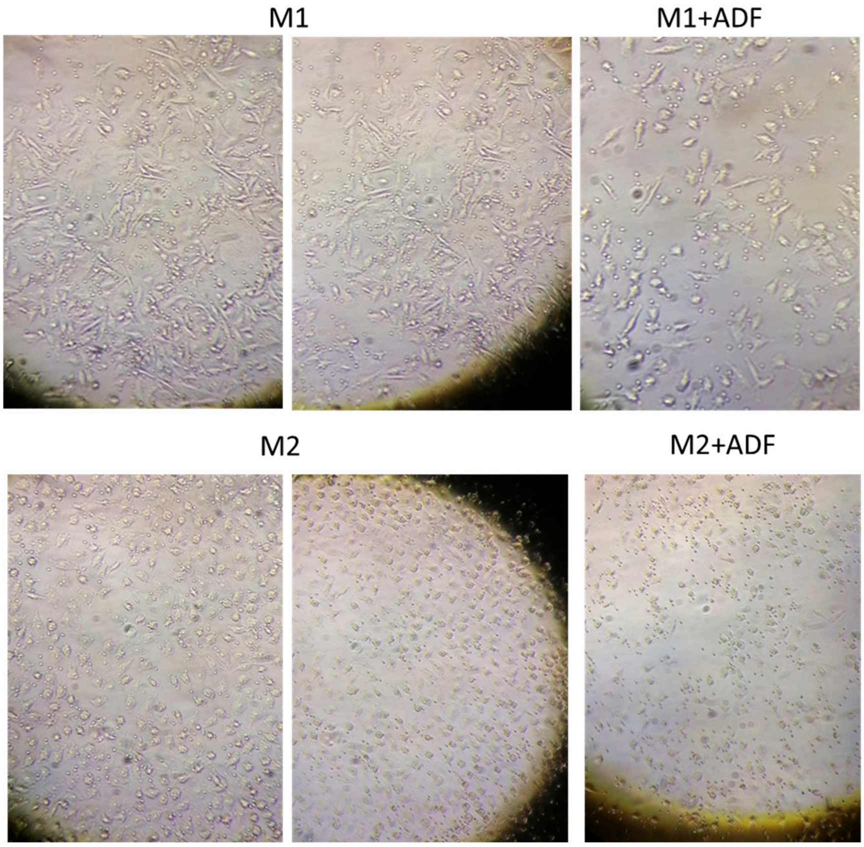

Figure S3. Photo of PBMC after various stimulation stages. M1 – cells were stimulated with M-CSF followed by stimulation with IFNγ and LPS; M1+ADF – cells were stimulated with M-CSF with sequential separate incubations with ADF, and then with IFNγ and LPS; M2 – cells were stimulated with M-CSF followed by stimulation with IL-4 and IL-13; M2+ADF - cells were stimulated with M-CSF with sequential separate incubations with ADF, and then with IL-4 and IL-13.
